# Chip-based *in situ* TEM investigation of structural thermal instability in aged layered cathode†

**DOI:** 10.1039/d3na00201b

**Published:** 2023-07-14

**Authors:** Yuhan Wang, Yuan Yuan, Xiaobin Liao, Gustaaf Van Tendeloo, Yan Zhao, Congli Sun

**Affiliations:** a State Key Laboratory of Advanced Technology for Materials Synthesis and Processing, International School of Materials Science and Engineering, Wuhan University of Technology Wuhan 430070 China yan2000@whut.edu.cn conglisun@whut.edu.cn; b Hongqi Integrated Circuit (Zhuhai) Co., Ltd China yuan@hq-nano.com; c NRC (Nanostructure Research Centre), Wuhan University of Technology Wuhan 430070 China; d EMAT (Electron Microscopy for Materials Science), University of Antwerp Belgium

## Abstract

Thermally induced oxygen release is an intrinsic structural instability in layered cathodes, which causes thermal runaway issues and becomes increasingly critical with the continuous improvement in energy density. Furthermore, thermal runaway events always occur in electrochemically aged cathodes, where the coupling of the thermal and electrochemical effect remains elusive. Herein, we report the anomalous segregation of cobalt metal in an aged LiCoO_2_ cathode, which is attributed to the local exposure of the high-energy (100) surface of LiCoO_2_ and weak interface Co–O dangling bonds significantly promoting the diffusion of Co. The presence of the LCO–Co interface severely aggregated the oxygen release in the form of dramatic Co growth. A unique particle-to-particle oxygen release pathway was also found, starting from the isolated high reduction areas induced by the cycling heterogeneity. This study provides atomistic insight into the robust coupling between the intrinsic structural instability and electrochemical cycling.

## Abstract 2

Thermally induced oxygen release is an intrinsic structural instability in layered cathodes, which causes thermal runaway issues and becomes increasingly critical with the continuous improvement in energy density.

## Graphical abstract

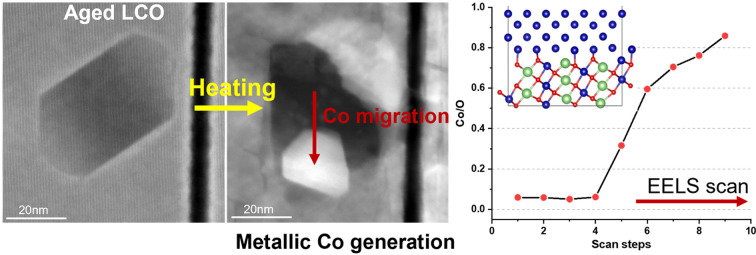

## Introduction

Layered lithium transition metal oxides (LTMOs) are the dominant cathode materials in lithium-ion batteries. Different from other cathode materials such as LiFePO_4_ and LiMn_2_O_4_, where their theoretical capacity has almost been reached, layered cathodes only exhibit slightly more than half of their capacities under actual working conditions. However, they still hold great potential for improving the energy density, and therefore, attracting increasing research interest.^1–5^ One of the general issues associated with layered cathodes is the release of the lattice oxygen, which contributes to a number of battery failures, including voltage/capacity fading,^6–9^ the loss of cations,^2,10^ increase in impedance,^11^ the cracking and detachment of primary particles.^10,12,13^ The released lattice oxygen reacts with the flammable carbon-based electrolyte, causing severe thermal runaway events. Furthermore, with the continuous improvement in the energy density, the thermal runaway issue has become increasingly critical, and thus the development of corresponding mitigating approaches is necessary. Various studies have reported the oxygen loss mechanism in layered cathodes.

Most studies assumed a homogenous and isotropic oxygen activity and degradation pathway, deriving averaged phase figures without considering the localization effect. This works well for uncycled single-crystal LiCoO_2_ (LCO) in which the thermal effect is dominant. However, the thermal runaway event always happens in electrochemically aged LCO and even during cycling, where secondary structures and complicated hierarchical surfaces/interfaces are also generally present. Consequently, the coupling of the thermal and electrochemical effect dominates the oxygen release. However, the underlying mechanism for the thermo-electrochemical coupling effect is still elusive.

Herein, a chip-based *in situ* heating experiment employing aberration-corrected scanning transmission electron microscopy (STEM) was performed to study the oxygen release behaviour in electrochemically aged LiCoO_2_. Voids were observed after extended cycling at a high cut-off voltage, which was induced from the activated oxygen redox and reduced oxygen ion migration barrier both in bulk and along the boundary. Anomalous metallic Co was found in the nanovoid, originating from the exposure of the high-energy (100) surface of LCO and weak surface Co–O dangling bonding, which significantly promoted the Co diffusion. The presence of the LCO–Co interface severely aggregated the oxygen release in the form of dramatic Co growth. A different pathway of oxygen release was also found, starting from the isolated high reduction area from the cycling heterogeneity. The degradation propagated in a particle-by-particle feature. This work provides atomistic insight into the robust coupling between the intrinsic structure instability and electrochemical cycling.

## Results and discussion

### Electrochemical cycling

**Fig. 1:**
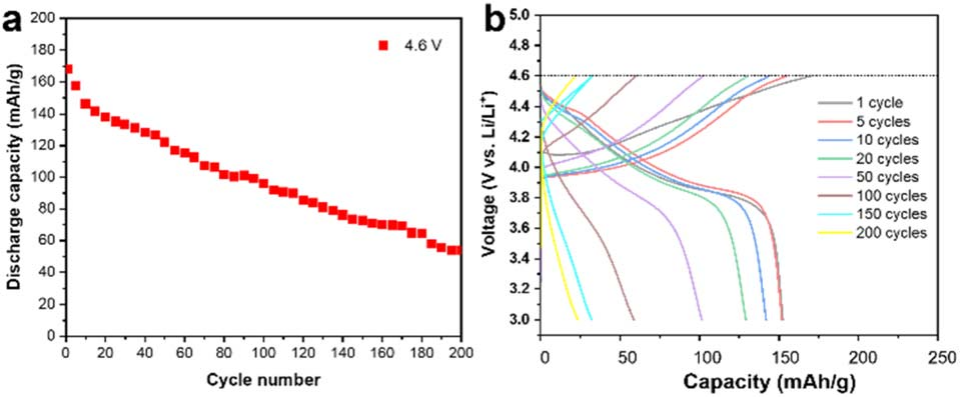
Electrochemical performance. (a) Specific capacity as a function of the cycling number. It shows the typical capacity fading of the cell using LCO as the cathode and Li metal as the anode with a high cut-off voltage of 4.6 V. (b) Charge–discharge profiles of the LCO cathode at different cycling numbers at 4.6 V.

A commercially available LCO cathode was used in this study, with the theoretical capacity of 178 mA h g^−1^, and its electrochemical performance is shown in Fig. 1 in the working voltage window of 3–4.6 V. The LCO cathode showed continuous capacity decay under 4.6 V at the current of 1 C and its capacity is only 50% of the initial capacity after 200 cycles, as shown in Fig. 1a. The corresponding charge/discharge curves for the cycling performance indicate the typical long-cycle instability and slight polarization of the commercial LCO cathode at a high cut-off voltage, as shown in Fig. 1b. It should be noted that polarization at high voltage cycling has been reported to be ubiquitous and may be caused by the cathode electrolyte interface (CEI), inhomogeneous charge distribution, choice of electrolyte and intrinsic structure of the cathode materials.^14,15^ The dense packing of LCO particles introduced a heterogeneous state of charge (SOC) and intricated interface/surface effects, which may contribute to the instability of LCO and capacity decay.^10,16,17^ It is believed that the side reactions between the LCO cathode and carbon-based organic electrolyte, especially at a high cut-off voltage, which results in the unstable highly oxidized state of Co^4+^ and O_2_^2−^, is the main reason for the failure of the LCO cathode.^18–20^

**Fig. 2:**
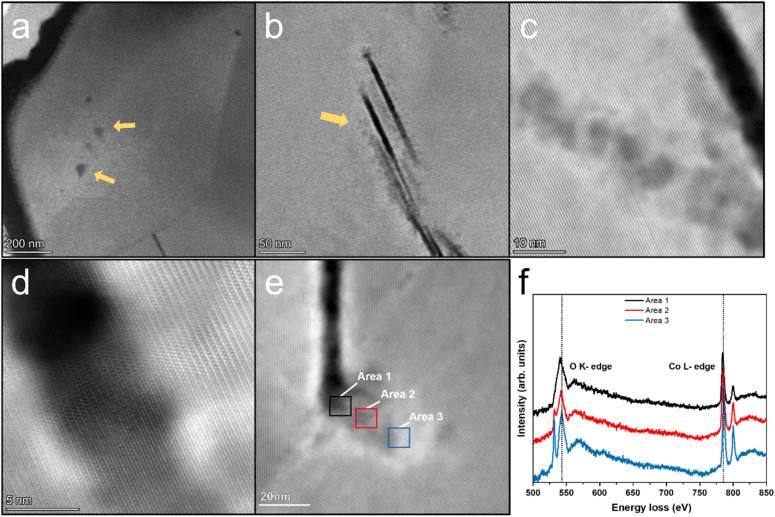
HAADF-STEM images of LCO cathode after 200 cycles at a high cut-off voltage of 4.6 V. (a) Low-magnification STEM image of LCO sample processed by FIB. Nanovoids are marked with yellow arrows. (b) Cracks inside the LCO grains are marked with yellow arrow. (c) Massive nanovoids exist near the crack. (d) Atomic-resolution STEM image of the crack end region. It shows the phase transition and stacking fault. (e) Crack ends at grain boundaries and structure degradation area adjacent to the crack. (f) EELS of O K-edge and Co L-edge of area 1, area 2 and area 3 marked in (e).

Fig. 2a shows high angle annular dark field (HAADF) STEM image of the cycled LCO. The electrochemical cycling caused various types of damage to the LCO particles such as phase transitions, dislocations, stacking faults and cracks, especially near the surface, thereby reducing the stability and cycling performance of the battery system,^21–24^ which is consistent with the STEM observations (shown as HAADF-STEM images in Fig. 1a–f in the ESI†). However, electrochemical cycling can also cause damage to the interior of the LCO particles. Generally, it is believed the bulk area of LCO is more stable than its surface region, which is susceptible to side reactions with the electrolyte,^25–27^ and the degradation is heterogeneous throughout the particle with a decreasing effect on the structure from the surface to the interior.^5,28,29^ However, the HAADF-STEM images of the LCO bulk in Fig. 2a–e show the local damage (marked by yellow arrows) from cycling, while the structure of the surrounding area remained intact, and this damage is easily produced at the grain boundaries. The STEM images of the pristine LCO before electrochemical cycling are shown in Fig. S2 in the ESI.† The results show that the pristine LCO maintained its layered structure without structural damage such as cracks, dislocations and phase transitions. The STEM images of the LCO cathode before and after beam irradiation are also presented in Fig. S3 in the ESI,† which indicates that no structural damage can be observed by the electron beam effect. We believe that the electrochemical cycling resulted in internal structural damage for several reasons. The first is the interior nanovoid induced by oxygen release. Given that the whole particle is involved in the charge/discharge process, oxygen is also involved in the redox reactions to provide extra capacity,^30^ and it is inevitable that the unstable high oxidation state of O_2_^2−^ still exists inside the stable bulk phase, which will give rise to the generation of oxygen gas. Due to the anisotropic properties of the secondary particle, which result in a heterogeneous state of charge,^10,31^ the oxygen redox is also inhomogeneous, where the oxygen atoms near the surface are more likely to be reduced and the possibility of oxygen release in the bulk area also exists. The second reason originates from the phase transition from the layered structure to the spinel and rock-salt structure on the surface of LCO due to the side reactions with the electrolyte, which creates a large number of oxygen vacancies, resulting in an oxygen vacancy concentration gradient and kinetically satisfying the oxygen vacancy diffusion from the surface to the interior. It is noteworthy that the oxygen vacancy is inclined to diffuse along the grain boundaries and at an accelerated rate.^32^ Thirdly, cracking from the surface to the interior provides a channel for the erosion of the electrolyte.^33,34^ While the reducing electrolyte encounters the highly oxidized state of Co^3+/4+^ and O_2_^2−^, the anion is reduced to generate oxygen gas. The structural degradation in the cracking-end region induced by oxygen release is shown in Fig. 2e. The corresponding EELS of the O K-edges and Co L-edges are shown in Fig. 2f. The oxygen pre-peak at 532 eV originates from the hybridization of the O 2p and Co 3d and 4sp states. The excitation of the O 1s orbitals to the unoccupied O 2p orbitals above the Fermi level gives rise to O K-edges in the EELS spectra. The hybridization reduced the number of unfilled O 2p states, which indicates Co valence states.^10,35^Fig. 2f reveals that the crack surface region has a lower oxygen content and the valence state of Co is also reduced in this area. This indicates that the exposed surface contains more oxygen vacancies.

### *In situ* heating of aged LCO

**Fig. 3:**
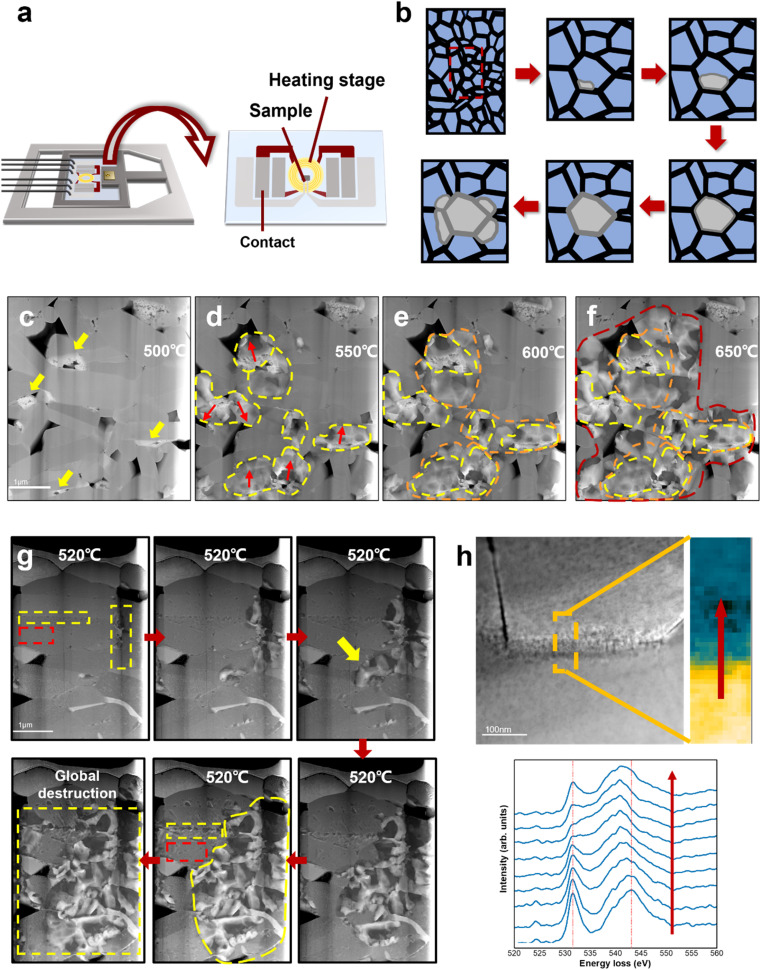
Thermal behaviour of LCO cathode. (a) Schematic illustration of the heating setup and the heating chip on the DENSsolution TEM holder. (b) Schematic illustration of the degradation process of electrochemically aged LCO cathode. (c)–(f) Degradation process of electrochemically aged LCO cathode at elevated temperature. The yellow arrows in (a) indicate the structurally damaged sites in the grain boundaries induced by high-voltage cycling. The regions marked by yellow, orange and red dash lines indicate the degradation propagation path. It shows that structural degradation in cycled LCO starts from the electrochemically damaged sites and spreads out to the entire grain, and eventually diffuses into neighbour grains, causing severe destruction to the whole LCO. (g) Degradation process of original LCO cathode. Areas marked by red rectangles are located in the LCO bulk, while areas marked by yellow rectangles are grain boundaries. Degradation from the grain boundaries is faster and more severe than from the interior, which starts from the growth of surface nanovoids (marked with yellow arrow). (h) Comparison of the O K-edge EELS of grain boundaries.

Fig. 3a shows a schematic illustration of the heating setup and the heating chip on the DENSsolution TEM holder. The TEM sample was connected to the chip contact by FIB processing, and the thermal field generated by the Mo coil could precisely control the sample temperature. The TEM sample was heated to 650 °C at a rate of 2 °C s^−1^. Fig. 3c–f show the HAADF STEM images of the same LCO particle taken at 500 °C, 550 °C, 600 °C, and 650 °C, respectively. The areas marked by the yellow arrows in Fig. 3c present the damage sites induced by electrochemical cycling, which existed before heating and usually aggregated on one side of the grain boundaries. The LCO sample remained intact until 500 °C, and then deteriorated rapidly after 500 °C. The red arrows in Fig. 3d indicate the direction of degradation propagation. The degradation spread out with the electrochemical damage sites as the core areas, and eventually propagated to the entire grain. It is noteworthy that these electrochemically damaged sites were mostly located at the grain boundaries or even multigrain junctions, which already had a high oxygen vacancy content, as shown by the EELS spectra of the O K-edges in Fig. 2f. The EELS spectra in Fig. 3h reveal that the Co ions in the grain boundary areas have a lower valence state than that in the interior regions, which implies an increasing loss of the neighbouring oxygen coordination and more oxygen vacancies in the grain boundaries. This difference in oxygen vacancy concentration gradients allowed the oxygen vacancy to migrate from the grain boundaries to the interior or diffuse along the grain boundaries, thus driving the structural degradation to propagate from the interfaces to internal areas. This is consistent with the observation in the electrochemical cycling. Given that some damaged regions appeared only on one side of the grain boundary, where the depletion of oxygen created a number of vacancies, it acted like a notch in the intact structure, and the degradation induced by heat accumulation could more easily directly swallow the layered structure inward from the damaged regions. The structural degradation was confined by the high-energy grain boundary instead given that it lacks enough energy to affect the adjacent grains at this time.

Interestingly, when the temperature was above 600 °C, the structural degradation was no longer restrained within the individual grain but crossed the grain boundaries and propagated to neighbouring grains (Fig. 3e and f). It is likely that the structural transformation lowered the migration barrier for oxygen vacancies to cross the grain boundary as a result of the aggravated grain boundary-oxygen release synergistic effect at elevated temperature, which broke the kinetic constrains of grain boundaries. More heat enabled the degradation to cross the grain boundary thermodynamically. A schematic illusion of the degradation process of electrochemically aged LCO cathode is shown in Fig. 3b. Meanwhile, the degradation rates at the surface/interface regions and bulk areas are different. The pristine LCO TEM sample was heated at 2 °C s^−1^ and started to deteriorate after 520 °C. In Fig. 3g, the red rectangle indicates the grain interior and the yellow rectangle indicates the grain boundary. The pristine LCO rules out the inhomogeneous state of charge, which suggests that the different degradation behaviours observed on the surface/interface and the bulk during heating can be attributed to the intrinsic structure of LCO. The degradation at the interface occurred earlier, and also propagated more rapidly compared to the grain interior. Given that the surface/interface has higher energy, the instability of its intrinsic structure makes oxygen escape more easily, and the resulting oxygen vacancy concentration gradient can further drive oxygen vacancy migrating to the grain interior. In addition, the threshold temperature also increased, indicating that more energy is required to destabilize the kinetic stability of the pristine LCO structure and trigger oxygen vacancy migration. Alternatively, the degradation occurs later in the bulk area at a much lower speed through the formation and growth of dispersive nanovoids (marked by red rectangle). Detailed nanovoid development with local phase dynamics was reported in our previous work.^20^ The structural degradation spreads out with nanovoids as the “catalysis” core has a low oxygen content, which is also the pathway of oxygen vacancy migration and oxygen release. Therefore, degradation at the interface causes more severe damage to LCO, and the characteristics of the surface/interface are an important factor in determining the cathode stability.

In summary, when an increase in temperature leads to thermal accumulation, the coupling effect of thermally induced degradation from the core areas introduced by the electrochemical cycling heterogeneity combined with the electrolyte-induced disintegration from the particle surface in battery operation will cause rapid failure of the LCO cathode. Therefore, it is significant to stabilize the bulk phase and improve the surface properties of LCO to enhance its stability.

### Co segregation

**Fig. 4:**
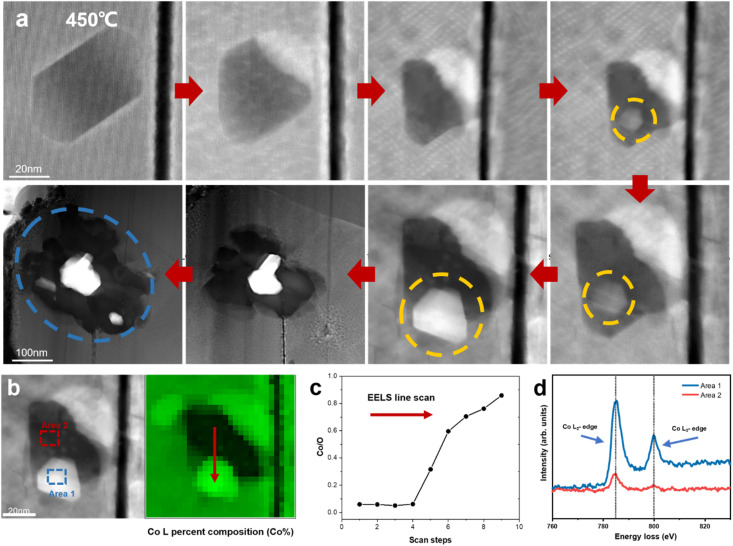
Anomalous generation of Co metal on LCO surface. (a) Dynamic tracking of the formation of Co metal in a regular void surface. (b) HAADF-STEM image of the void. (c) Co/O ratio from the EELS scan in the void. (d) Comparison of EELS of Co L-edges in area 1 and area 2 marked in (b).

Previous research has demonstrated the dissolution of transition metals in layered cathodes, which is closely related to the cathode instability and capacity decay due to the direct contact between the aqueous electrolyte and the cathode surface.^36,37^ Similarly, the dissolution of cobalt was observed during *in situ* heating, while the anomalous formation of metallic Co was also revealed. Fig. 4a depicts the degradation process of a regular hexagonal void with the precipitation of Co. The void starts to deteriorate from 450 °C and Co gradually grows in the void (region shown by the yellow circle in Fig. 4a). The EELS spectra shown in Fig. 4b and c demonstrate that this region is almost pure Co metal with the Co/O ratio almost reaching 0.9. Meanwhile, the comparison of the Co L-edges between the void interior and the void surface indicates that Co migrates from the inside of the void to the surface. Our previous work also provides strong evidence that this region is oxygen poor (Co rich) and the formation of amorphous Co nanospheres was also observed in the void.^20^ Previous research has reported that an irreversible phase transition from the rhombohedral to monoclinic phase results in the dissolution of Co.^36,38^ However, according to our observation, the edge of the void remained sharp and clear and the surrounding LCO structure was stable without apparent changes. It has also been reported that particle cracks may expose free surfaces, which assist the migration and dissolution of Co.^39,40^ Similarly, the void inside the particle is not directly connected with the cracks. Although a crack existed on the right side of the void, Co metal did not grow on the exposed surface in the crack. Facet-dependent segregation of the transition metal was also revealed given that Co tended to segregate to the (104) facet, as well as the dissolution of Co on the (001) face in LCO.^37^ However, these studies focused on the cathode-electrolyte interface, where Co either dissolves in the electrolyte or deposits on the CEI, thus increasing the cell resistance and reducing the electrochemical performance of the cathode materials, while no systematic and universal elaboration of Co migration and aggregation behaviour inside the particles and grains has been reported to date.

**Fig. 5:**
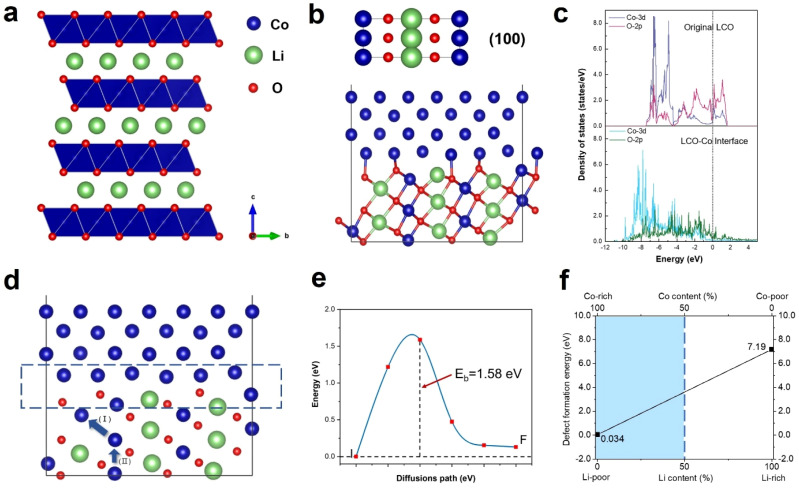
Density functional calculation of the formation of Co metal. (a) Schematic illustration of LCO structure. (b) LCO–Co interface model based on the LCO (100) facet. (c) Local density of states of Co 3d orbitals and O 2p orbitals of the pristine LCO (above) and LCO–Co interface (below). (d) Schematic illustration of Co co-migration path. Region marked by blue rectangle indicates the interface of LCO and Co. (e) Co migration barrier of path (II) in (d) calculated by the climbing image-nudged elastic band method. (f) Formation energy of oxygen vacancy as a function of Li content.

Density functional theory (DFT) calculations were performed to understand the anomalous generation of Co metal. The void may expose certain high-energy facets, which are usually hidden but favours Co migration. The three typical stable and non-polar facets of (100), (110) and (104) of LCO were considered. The LCO (100)–Co interfacial model is shown in Fig. 5b, which is the most stable model and has the lowest interfacial formation energy of 2516 mJ m^−2^. According to Table 1 in the ESI,† the (100) facet has the highest surface energy, which suggests that it is difficult to be exposed in most cases, whereas the LCO–Co model favours it given that the Co metal is stable on the LCO (100) surface and the (100) facet may promote the migration of Co. The void is oxygen poor, which indicates the presence of a large number of oxygen vacancies. The oxygen vacancy formation energy was calculated by the DFT method, as shown in Fig. 5f, which drops sharply at the Co-rich and Li-poor region to 0.034 eV. Two extreme cases, namely Co-rich (Li-poor) case and Co-poor (Li-rich) case, were calculated. The blue area in Fig. 5f is the range of oxygen vacancy formation energy when the Li content is below 50%. Given that the LCO sample is in the delithiated state and the cobalt content at LCO–Co interface area increases sharply, it can be considered that the LCO–Co interface is rich in cobalt and poor in lithium, in which case, the formation energy of oxygen vacancies can be as low as 0.034 eV. This suggests that oxygen vacancy is easily generated at the interface and the diffusion of oxygen vacancies can continuously break the kinetic stability of the layered structure, and ultimately massive oxygen vacancies may break the stable CoO_6_ octahedral structure, in which case, Co is more likely to escape from the CoO_6_ octahedron without the constraint of Co–O bonding. The hybridization of O 2p orbitals and Co 3d orbitals decreases at interface region according to the local density of states (LDOS) of the pristine LCO and LCO–Co interface, as shown in Fig. 5c. Due to the absence of sufficient oxygen, the breakage of the Co–O bond may disrupt the CoO_6_ octahedral bonding or cause distortion of the CoO_6_ octahedra, which further decreases the Co migration barrier. The results showed that Co metal is oxyphilic, and the detached oxygen atoms are easily combined to form oxygen gas.^41,42^ With the increasing absence of Li and O atoms in the LCO structure, the remaining Co atoms aggregate and grow more rapidly, swallowing up the surrounding structure. Fig. 4a also shows that the formation of Co metal is extremely destructive to the local structure, which will eventually affect the global structure of LCO (marked by blue circle in Fig. 4a).

The anomalous Co metal growth indicated a nucleation-growth process. The Co cross-layer migration is considered to be a possible route for Co dissolution in layered cathode materials.^40^ The DFT calculation suggests that Co will spontaneously migrate to the Li sites assisted by oxygen vacancies, as shown as path (I) in Fig. 5d. The octahedral Li/Co cation vacancies drive Co to occupy the tetrahedral sites to compensate for the extra negative charge.^37^ The LCO–Co interfacial model also displays a Co migration channel through which Co can achieve easier interlayer movement, resulting in the fact that the Co atoms in the bulk area can be enriched at the near-surface sites. When Co atoms further accumulate on the void surface, the surface tension exceeds the Coulomb repulsion of surface Co to internal Co, and Co will nucleate and propagate in an almost oxygen-free environment.

Besides, the migration of Co to Li sites induced by oxygen vacancies is demonstrated to be possible, but the removal of a Co atom leaves a Co vacancy with negative charges, which will break the equilibrium of the adjacent CoO_6_ octahedra. Therefore, the Co atom below may gain extra energy to jump to the above Co vacancy, which is presented as path (II) in Fig. 5d, and the migration barrier energy calculated by the CL-NEB method is shown in Fig. 5e. This co-migration process provides a constant source of Co for the growth of Co metal. Compared to the general circumstance where the migration barrier of Co is 2.33 eV,^32^ the migration barrier of Co in the co-migration case is only 1.58 eV, which reveals a highly reduced Co kinetic stability by compromising the trapping strength of the CoO_6_ framework.

There are many other factors that affect the migration barrier of Co atoms and Co metal formation. Here, we discussed one possible reason through DFT calculations. It should be noted that once Co dissolves and forms Co metal on the surface of LCO, it will cause severe and global destruction of the LCO cathode, and thus the LCO structure will collapse dramatically (marked with blue circle in Fig. 4a), which will seriously threaten the stability and safety of the battery. Therefore, suppressing the generation of oxygen vacancies and the cycling heterogeneity is a significant way to improve the stability of LCO.

## Conclusions

The coupling effect of electrochemical cycling and thermal abuse was systematically investigated. The *in situ* TEM heating experiments demonstrated a unique particle-to-particle thermal degradation pathway in LCO at the atomic scale, which is closely associated with the heterogeneous oxygen vacancy kinetics and thermodynamics at elevated temperature. The LCO interior damage sites introduced by electrochemical cycling contained a lower oxygen content, which resulted in a difference in oxygen vacancy concentration gradient, driving the migration of oxygen vacancies towards the whole grain. The degradation propagated along the pathway of oxygen release with isolated reduced damage sites as the core areas, firstly within a single grain due to the constraint of grain boundaries, and then degradation crossed the grain boundaries, spreading to adjacent grains once the thermodynamics broke the kinetic stability at elevated temperature. An anomalous Co segregation in the aged LCO surface was observed during heating, which is attributed to the local exposure of the high-energy (100) surface of LCO and weak interface Co–O dangling bonds in a void, which significantly promoted the diffusion of Co. Oxygen vacancies were easily formed in the extremely cobalt-rich region (the oxygen vacancy formation energy was reduced to 0.034 eV), and the stability of the CoO_6_ octahedron was reduced by weakening the Co–O bonding at the LCO–Co interface, which favoured the co-migration of Co atoms from the bulk interior to the surface, providing a continuous cobalt source for the rapid growth of Co metal. This study sheds light on the underlying mechanism of the thermal stability of aged LCO.

## Methods

The *in situ* STEM samples were prepared using an FEI Helios Nanolab G3 dual-beam focused ion beam (FIB) and the standard lift-out procedure following our previous publication.^20,32^ The cycled LCO cathode sample was dissembled from a half-cell and ultrasonically cleaned with alcohol to remove the residual electrolyte. The original LCO sample was not subjected to electrochemical cycling. The sample was deposited with e-beam- and ion-beam-induced Pt deposition on the surface inside the FIB chamber. Then, the sample was transferred to a DENS solution heating chip, polished by ion beam with several times to a specific TEM sample thickness at the target area. The SiN_*x*_ chip-supporting film was milled to open a window for better observation.

The STEM images and EELS spectra were acquired using a double-spherical aberration-corrected high-resolution transmission electron microscope (CEOS probe corrected TEM, Titan Themis, FEI), at an electron accelerating voltage of 300 kV with a probe convergence angle of 17.8 mrad, spatial resolution of 0.08 nm, probe current of ∼30 pA for STEM imaging and ∼100 pA for EELS acquisition, and EDS energy resolution of 136 eV with an effective detection area of 120 mm^2^. The inner semi-angular angle for the HAADF detector was 84 mrad for STEM imaging. EELS was performed using a Gatan Quantum 965 GIF system with an energy resolution of 0.25 eV. Dual EELS was acquired with a spectrometer dispersion chosen for simultaneous visualization of zero loss and O K and Co L edges. The energy resolution determined by full-width at half-maximum of the zero-loss peak was ∼1.3 eV. The background was subtracted using the power law method, with plural scattering removed by Fourier-ratio deconvolution. The EELS spectra herein were normalized.

DFT calculations were performed using VASP (Vienna *Ab initio* Simulation Package). The electronic exchange-correlation function employed the Perdew–Burke–Ernzerhof (PBE) generalized gradient approximation (GGA). The GGA + U method was used for all calculations to correct the strong electronic correlation among the localized Co 3d electrons with the U value set as 3.91 eV for Co. An energy difference was set to 1.0 × 10^−7^ eV per atom to ensure the accurate electronic ground-state calculation. The maximum force tolerance was set to 0.02 eV Å^−1^ for structural optimization. The energy cut-off for the plane-wave basis expansion was set to 520 eV. The *k*-points for Brillouin zone were selected by the Gamma method and set to 3 × 3 × 1 for original LCO structural optimization and 3 × 3 × 2 for LCO–Co interface model optimization. The migration barrier of Co atoms in the LCO–Co system was obtained by the climbing image nudged elastic band (CI-NEB) method with 4 climbing images between the initial and final states. The LCO–Co interface model was built based on the LCO (100) surface and the Co (110) surface with a vacuum thickness of 10 Å by Materials Studio. The lattice mismatch was around 10% of the LCO–Co interface model but was eliminated after structural optimization. Local density of state (LDOS) calculation was also performed for the original LCO and LCO–Co interface model.

A commercial LiCoO_2_ (LCO) cathode was used for this study. The electrochemical tests were performed using CR2016 coin cells, which were assembled with lithium metal foil as the anode and the cathode electrodes were composed of 80% active material (LCO), 10% acetylene black and 10% polyvinylidene fluoride (PVDF) coated on Al foil. The electrolyte was composed of EC, DEC and DMC (1 : 1 : 1 ratio/volume) as a mixed solution. Galvanostatic charge/discharge tests were performed by using a LAND CT2001A multichannel testing system. The electrochemical cycling was performed in the voltage range of 3–4.6 V (*vs.* Li^+^/Li) at the current of 1 C. The coin cells were disassembled after 200 cycles in the discharge state. All the assembly and disassembly processes were performed in a glovebox filled with pure argon gas. The cathode material was immersed in a pure DMC solution before cleaning and loading on an Al_2_O_3_ stage for FIB processing.

## Author contributions

C. S. and Y. W. conceived the project. Y. Y. designed the *in situ* chip contact setup. C. S. and Y. W. performed the *in situ* TEM and interpretation. Y. W., X. L. and Y. Z. conducted the DFT simulations. Y. W. conducted the battery measurement and data interpretation. All the authors contributed to the writing of the manuscript before submission.

## Conflicts of interest

There are no conflicts to declare.

## Supplementary Material

NA-005-D3NA00201B-s001
