# Supplementary material for: Chip-based in situ TEM investigation of structural thermal instability in aged layered cathode
Source: Nanoscale Adv. 2023 Jul 14;5(16):4182–90. doi: 10.1039/d3na00201b (PMC10408578; doi:10.1039/d3na00201b)
Supplement: NA-005-D3NA00201B-s001 [file NA-005-D3NA00201B-s001.pdf]

Electronic Supplementary Material (ESI) for Nanoscale Advances.
This journal is © The Royal Society of Chemistry 2023

(Supplementary Materials)

Chip-based in situ TEM Investigation of Structural Thermal Instability in Aged Layered Cathode

Yuhan Wang[1], Yuan Yuan[2]*, Xiaobin Liao[1], Gustaaf Van Tendeloo[3,4], Yan Zhao[1]*, Congli Sun[1,3]*

[1]State Key Laboratory of Advanced Technology for Materials Synthesis and Processing, International School of Materials Science and Engineering, Wuhan University of Technology, Wuhan 430070, China

[2]Hongqi Integrated Circuit (Zhuhai) Co., Ltd., China

[3]NRC (Nanostructure Research Centre), Wuhan University of Technology, Wuhan 430070, China

[4]EMAT (Electron Microscopy for Materials Science), University of Antwerp, Belgium

*Corresponding authors: yuan@hq-nano.com. yan2000@whut.edu.cn. conglisun@whut.edu.cn.

This file includes

S1. STEM characterizations

S2. LCO-Co interface model

S3. Oxygen vacancy formation energy

S1 STEM characterizations

A half-cell composed of commercial LCO cathode was disassembled after 200 cycles under the voltage of 3-4.6 V at the current of 1C in a glovebox filled with pure argon gas. The atomic images characterized by double spherical correction TEM show that, electrochemical cycling at high voltage will cause severe structural degradation to LCO. Fig. S1a shows a low magnification image of LCO sample with apparent structure damage. Fig. S1b presents intergranular cracks and intragranular cracks which are likely to appear around grain boundaries and may result in electrolyte dissolution and erosion. Various mechanisms have been reported to account for the formation of cracks such as inhomogeneous strain, phase transition and oxygen release and so on[1-4]. Fig. S1c shows phase transition at crack surface and extra contrast introduced by Co atoms migrates to tetrahedral vacancies which indicates the onset of phase transition. Fig. S1d shows a stacking fault in layered structure. Fig. S1e shows one end of the crack where exists a clear phase transition from layered structure to spinel structure. It is believed nanovoid is closely related to the generation of cracks[2, 5]. Fig. S1f presents LCO surface area and surface reconstruction can be observed, which may be cause by the direct contact with reductive carbon-based electrolyte. Fig. S2 shows the STEM characterizations of pristine LCO before cycling. It demonstrates that the pristine LCO maintains layered structure, without structural damages such as cracks, dislocations, and phase transitions, and all the structural damages are induced by electrochemical cycling. Figure S3 shows the STEM images of LCO cathode before and after beam irradiation. The Figure S3b presents indicates that LCO maintains

layered structure without any damage after beam irradiation for 30 minutes at room temperature.

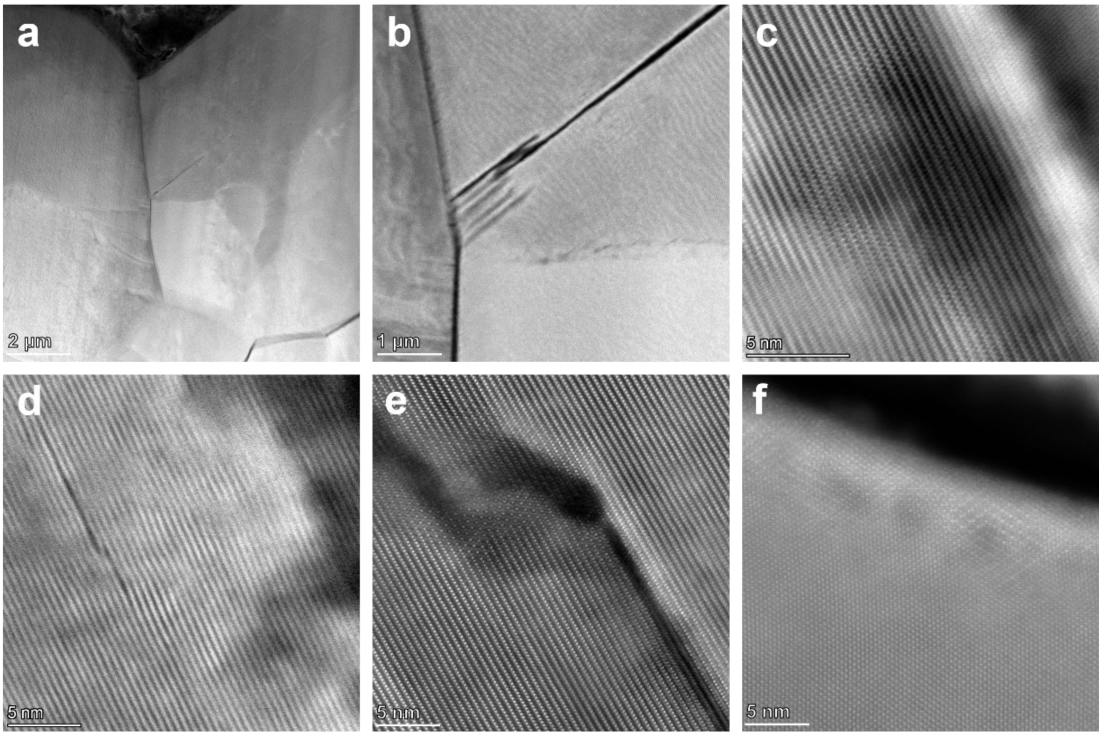

Figure S1 HAADF-STEM images of cycled LCO. **a**, Low-magnification image of densely packing LCO. **b**, Intergranular cracks and intragranular cracks introduced by electrochemical cycling are apparently dispersed in LCO grains. **c**, Regions near crack surface, the Co atoms occupy the tetrahedral sites which shows the beginning of a phase transition. **d**, The stacking faults in the layered structure. **e**, The epitaxial phase transition near crack and void. A clear spinel phase can be observed. **f**, Surface of LCO which shows surface reconstruction and phase transformation.

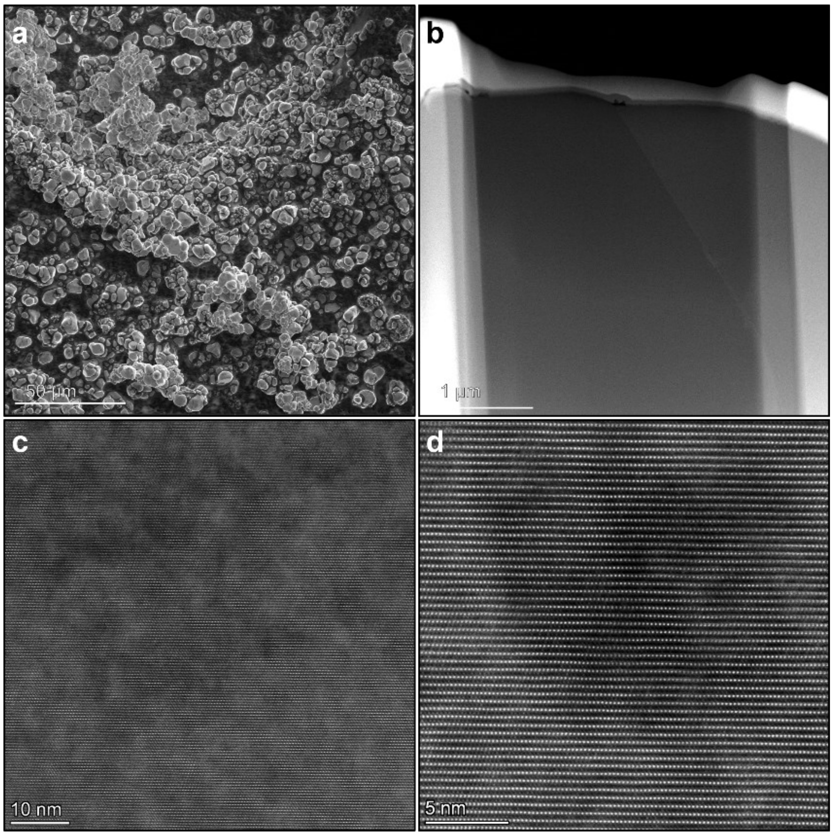

Figure S2 The STEM characterizations of pristine LCO before cycling. **a,** SEM images of pristine LCO particles. **b**, Low magnification image of pristine LCO TEM sample processed by FIB. **c**, High magnification image of pristine LCO cathode. The layered structure without damage is clear. **d**, The STEM image at atomic scale of layered structure of pristine LCO.

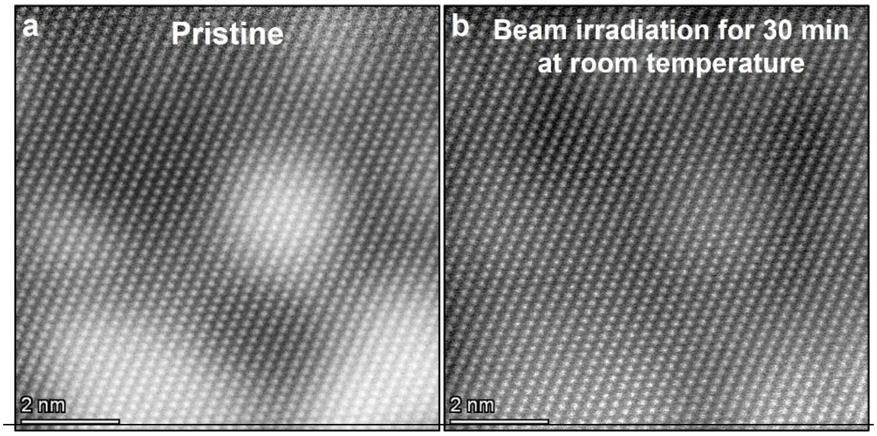

Figure S3 The STEM images of layered LCO cathode before (a) and after (b) beam irradiation. The same amount of beam irradiation was taken for 30 minutes at room temperature. The results show that the layered structure is maintained well.

S2 LCO-Co interface model

Various facets of LCO have been explored, and three nonpolar facets were considered in this paper because other polar facets of LCO appear in a highly oxidized or reduced environment which have instable chemical properties. The surface properties of three facets are presented as Table S1 and Fig. S4. The lattice mismatch of LCO-Co interface model is around 10% but was eliminated during structural optimization. The conjugated and non-conjugated LCO-Co interface model were also considered.

We first built LCO 3×2×1 supercell and Co 3×2×1 supercell, and according to the surface energy calculation equation (1) in VASP,

$$\sigma = \frac{1}{2A}\left(E_{slab} - N \cdot E_b\right) \tag{1}$$

Where σ indicates the surface energy, A is the surface area in the supercell, E_{slab} is the total energy of slab, N is the number of atoms in the slab and E_b is the bulk energy per atom. We could get the energy of different LCO and Co surfaces by building different LCO-Co interfacial model which are presented as Fig. S5, and the LCO (100) - Co interfacial model is the most stable one and has the lowest interface formation energy as 2516 mJ/m^2.

**Surface**	**u**	**v**	**a[Å]**	**b[Å]**	**∠(a,b)[deg]**	**coord.**	**γ[mJ/m^2]**
(100)	[010]	[001]	2.802	14.089	90.0	3/5	2943
(110)	[001]	[1-10]	14.089	4.853	90.0	4/6	2241
(104)	[42-1]	[010]	17.109	2.082	90.0	5/6	1048

Table S1 The surface energy of (100), (110) and (104) facets of LCO based on which LCO-Co interface model were built[5].

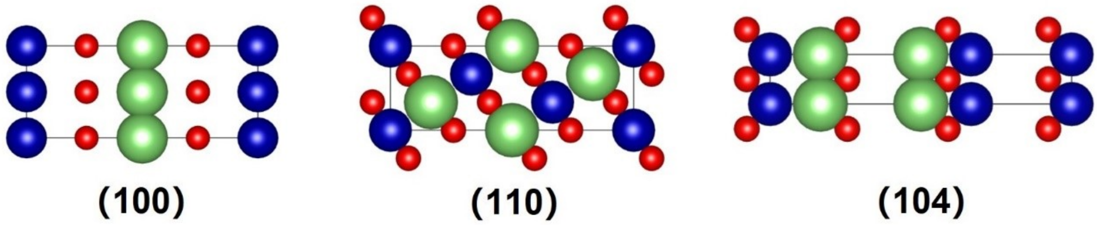

Figure S4 The schematic illustration of LCO (100), (110) and (104) surface, respectively.

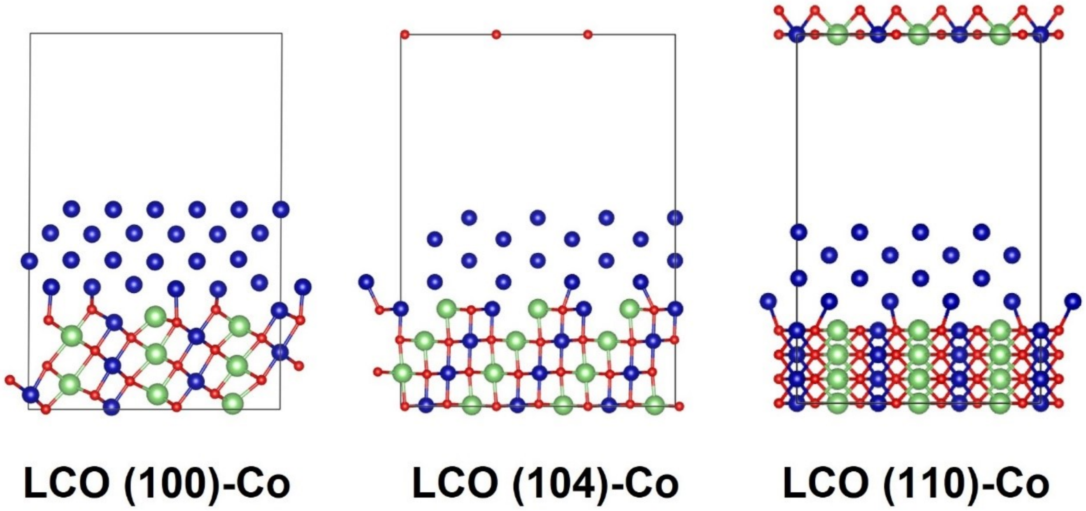

Figure S5 The schematic illustration of LCO (100)-Co interfacial model, LCO (110)-Co interfacial model and LCO (104)-Co interfacial model. The LCO (100)-Co interface had the lowest interface energy by DFT calculations.

S3 Oxygen vacancy formation energy

Oxygen vacancy formation energy was calculated by DFT methods. The region at the interface can be considered as lithium-poor and cobalt-rich. We consider two extreme cases, i.e., Co-rich (Li-poor) conditions and Co-poor (Li-rich) conditions. Then the oxygen vacancy formation is in the range of these two conditions.

Under Co-rich and Li-poor conditions, the oxygen vacancy formation energy can be calculated from equation (2),

$$E^f\left(V_O^{2+}\right) = E_{tot}\left(V_O^{2+}\right) - E_{tot}(LCO) + \mu_O + \mu_{Co} \tag{2}$$

Under Li-rich and Co-poor conditions, the oxygen vacancy formation energy can be calculated from equation (3),

$$E^f\left(V_O^{2+}\right) = E_{tot}\left(V_O^{2+}\right) - E_{tot}(LCO) + \mu_O + \mu_{Li} \qquad (3)$$

Where μ_O is set to an upper bond given by the energy of an O atom in an O_2 molecule.

LCO interfacial model contains three types of oxygen atoms with different chemical environment (marked as atom 1, 2, and 3 with different colors). Oxygen vacancy formation energy was considered in Co-rich (Li-poor) case and Co-poor (Li-rich) case[6].

The blue region in Fig. 5f in the article is the range of oxygen vacancy formation energy below 50% of Li content. Since the LCO sample is in the delithiated state, the cobalt content at the interface increases sharply, so it can be considered that it is almost in the ideal state of Co-rich and Li-poor at the LCO-Co model interface, so the oxygen vacancy formation energy can be as low as 0.034 eV under this condition.

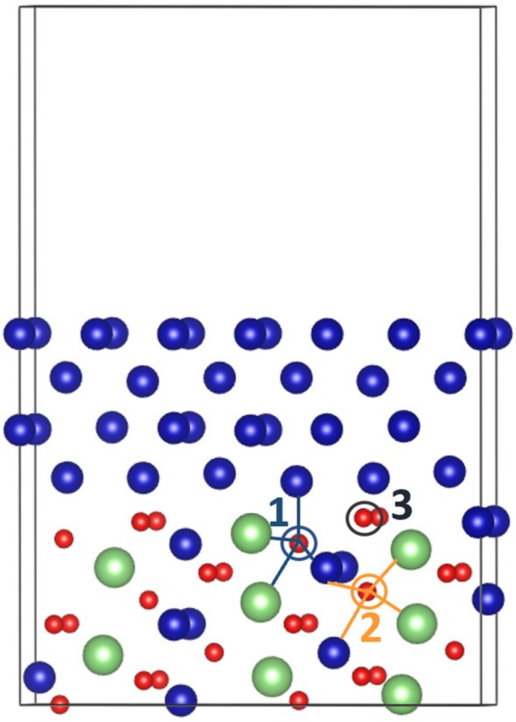

Figure S6 Oxygen vacancy formation energy of three types of oxygen atoms with different chemical environment (marked as atom 1, 2, and 3 with different colors) was calculated by the DFT methods as 0.034 eV, 0.047eV and 0.039 eV, respectively, at the

Co-rich case.

References

1. S. Y. Yin, W. T. Deng, J. Chen, X. Gao, G. Q. Zou, H. S. Hou and X. B. Ji, *Nano Energy*, 2021, **83**, 37.
2. M. M. Besli, S. H. Xia, S. Kuppan, Y. Q. Huang, M. Metzger, A. K. Shukla, G. Schneider, S. Hellstrom, J. Christensen, M. M. Doeff and Y. J. Liu, *Chem. Mat.*, 2019, **31**, 491-501.
3. Q. Y. Lin, W. H. Guan, J. B. Zhou, J. Meng, W. Huang, T. Chen, Q. Gao, X. Wei, Y. W. Zeng, J. X. Li and Z. Zhang, *Nano Energy*, 2020, **76**.
4. J. H. Kim, H. H. Ryu, S. J. Kim, C. S. Yoon and Y. K. Sun, *Acs Applied Materials & Interfaces*, 2019, **11**, 30936-30942.
5. D. Kramer and G. Ceder, *Chem. Mat.*, 2009, **21**, 3799-3809.
6. X. Y. Chong, P. W. Guan, Y. Wang, S. L. Shang, J. P. S. Palma, F. Drymiotis, V. A. Ravi, K. E. Star, J. P. Fleurial and Z. K. Liu, *Acs Applied Energy Materials*, 2018, **1**, 6600-6608.
